# Circulating PD-1^+^ effector memory T cells predict anti-PD-1 efficacy in advanced gastric cancer

**DOI:** 10.3389/fimmu.2025.1720724

**Published:** 2025-12-10

**Authors:** Kongcheng Wang, Jie Shao, Kai Xin, Lianru Zhang, Baorui Liu, Wenxiu Chen, Qin Liu

**Affiliations:** 1 Department of Oncology, Nanjing Drum Tower Hospital, Affiliated Hospital of Medical School, Nanjing University, Nanjing, China; 2 Clinical Cancer Institute of Nanjing University, Nanjing, China

**Keywords:** effector memory T cells, PD-1, immunotherapy, biomarker, gastric cancer, peripheral blood

## Abstract

**Background:**

Although standard chemotherapy combined with immunotherapy represented by programmed cell death -1(PD-1) blockade shows promise as a treatment approach for gastric cancer (GC), the drug response rates remain low. Identifying reliable biomarkers to predict patient response is urgently needed.

**Methods:**

We collected baseline (before receiving PD-1 inhibitors) peripheral blood samples from advanced GC patients (n=54) who underwent PD-1 inhibitors and platinum drugs from January 2022 to December 2023 and investigated PD-1 expression on effector memory T (TEM) between responders and non-responders according to Response Evaluation Criteria in Solid Tumors (RECIST 1.1) guidelines by low-dose computed tomography scan evaluation. Univariate and multivariate analyses were applied to identify potential predictive biomarker for chemo-immunotherapy combinations outcomes. *In vitro* cytotoxicity and cytokine detection were used to demonstrate the effects of PD-1 blockade on TEM cells with different levels of PD-1 expression.

**Results:**

The optimal cutoff values for percentages of PD-1^+^ cells in TEM were 8.66%. Among advanced GC patients receiving anti-PD-1 therapy, responders exhibited a higher mean percentage of PD-1^+^ cells in TEM (*P* = 0.017) than non-responders, which were associated with longer PFS. Univariate and multivariate Cox regression analyses demonstrated that higher percentage of PD-1^+^ cells in TEM (HR = 0.191, 95% CI 0.065–0.560, *P* = 0.003) was independent protective factor in advanced GC patients receiving chemo-immunotherapy. *In vitro*, PD-1 blockade enhanced TEM activation, as evidenced by increased cytotoxicity and IFN-γ secretion, leading to improved anti-tumor capacity.

**Conclusion:**

Our data identify a high pretreatment percentage of PD-1^+^ cells in TEM as a potential predictive biomarker for response to PD-1 blockade.

## Introduction

Gastric cancer (GC) is one of the most aggressive and deadly forms of human cancer, ranking among the top five malignant tumors globally in terms of both incidence and mortality rates (1–3). Despite chemotherapy being the routine treatment for advanced GC, the patient population that experiences clinical benefit is quite restricted. The median overall survival (OS) for advanced-stage GC treated with conventional chemotherapy is less than one year (4, 5). The emergence of immune checkpoint inhibitors (ICI) therapy, has led to extensive research indicating that the combination of chemotherapy and immunotherapy yields far greater effects than chemotherapy monotherapy. In the cohorts 2 and 3 of the phase II KEYNOTE-059 trial, which consists of 25 patients with advanced GC showed that patients treated with pembrolizumab plus 5-fluorouracil and cisplatin achieved a higher objective response rate (ORR) than patients treated with pembrolizumab monotherapy (6). Nevertheless, due to the complexity of the tumor microenvironment, ICI therapy faces frequent resistance in actual clinical practice, underscoring the urgent need for new targets to predict the precision of immunotherapy, highlighting the pressing challenge of screening and selecting the suitable patient population (7). Certain tissue-based biomarkers, such as programmed cell death-ligand 1(PD-L1), defective mismatch repair (MMR) and tumor mutational burden (TMB), have seen widespread use in screening populations that may benefit from ICI therapies (8–10). Given the limited availability of tumor samples, more readily accessible biomarkers such as peripheral blood samples should be evaluated for routine clinical application.

Memory T cells can provide long-term immunity, which are ultimately responsible for effective immune surveillance. Tissue-resident memory T (TRM) cells have been shown to be a potent predictive biomarker of the response of cancer patients to ICI therapies in various cancers such as lung cancer, breast cancer and gastric cancer (11–14). Additionally, the indicative significance of peripheral blood memory T cell for ICI therapies in esophageal cancer and gastric cancer has also been documented (15, 16). Since the anti-PD-1 antibody binds to cells expressing PD-1, identifying the specific immune cell types that interact with the anti-PD-1 antibodies could help predict which patients may respond best to anti-PD-1 therapy. In view of the unmet predictive value of memory T cells for the efficacy of ICI therapies, we hypothesized the expression profile of PD-1 on diverse memory T cell populations may display disparities, and we investigate its prospective utility as a predictive indicator in our research.

## Materials and methods

### Patients and study design

All specimens and relevant clinical data were obtained from the Comprehensive Cancer Center of Nanjing Drum Tower Hospital. The baseline clinical characteristics, as well as peripheral blood routine indices, biochemical markers, immunological indicators, and PD-L1 CPS scores, were all extracted from electronic patient records and the study was approved by the Ethics Committee of The Affiliated Nanjing Drum Tower Hospital, Medical School of Nanjing University (Nanjing, China) (No. 2023-459-01). Only patients who agreed to the study and provided signed consent were included. Further inclusion criteria were as follows: (1) Patients over 18 years old, (2) Patients with histopathological diagnosis of GC, at a locally advanced or metastatic stage, (3) Patients received PD-1 inhibitors plus chemotherapy (platinum drugs) at least twice and at least had a post-baseline computed tomography scan, (4) Patients with complete evaluable peripheral hematological parameters and peripheral blood samples before the initiation of PD-1 inhibitors. A total of 54 patient cases were gathered, including their pre-treatment clinical data, peripheral blood samples as well as imaging data, and these variables were subsequently evaluated in accordance with the treatment response. Baseline measurements were defined as those taken before receiving PD-1 inhibitors.

### Evaluation of efficacy

Low-dose computed tomography scan was performed before treatment and every 2 cycles of treatment, the best overall response was assessed using the Response Evaluation Criteria in Solid Tumors (RECIST 1.1) guidelines (17). Responses were classified as follows, including complete remission (CR), partial remission (PR), stable disease (SD), and progressive disease (PD). Progression-free survival (PFS) refers to the time from the start of treatment to disease progression or death from any cause. CR, PR and SD for >6 months were defined as responders; PD and SD for <6 months, were defined as non-responders (18).

### Peripheral blood immune parameters determination

First, EDTA-anticoagulated whole blood was stained with APC-conjugated CD3 (UCHT1, Beckman Coulter, USA), PE-conjugated CD45RO (UCHL1, Beckman Coulter, USA), FITC-conjugated CD62L (DREG-56, Biolegend, USA), and PE-Cyanine7-conjugated PD-1 (PE-Cyanine7, Biolegend, USA) antibodies for 20 minutes according to the manufacturer’s instructions. Red blood cell lysis buffer was then added and incubated for 15 minutes, followed by two washes with phosphate buffer saline (PBS). After washing, cells were resuspended in PBS. Acquisition was performed on CytoFlex flow cytometry (Beckman Coulter). Data analyses were performed with CytExpert software. The proportion of each marker is analyzed from the lymphocyte gate.

### Cells and culture medium

Human gastric cancer cell line MKN45 were purchased from the Cell Bank of Shanghai Institute of Biochemistry and Cell Biology and cultured in Roswell Park Memorial Institute (RPMI) 1640 supplemented with 10% FBS (Gibco, USA), 1% penicillin-streptomycin.

### Generation and characterization of TEM cells

PBMCs were collected from three GC patients with sufficient quantities of cryopreserved PBMCs from the 54 enrolled patients using Ficoll density centrifugation. For purification of TEM, human PBMCs were stained for CD3 (UCHT1, Beckman Coulter, USA), CD45RO (UCHL1, Beckman Coulter, USA) and CD62L (DREG-56, Biolegend, USA). Live, single cells were sorted into TEM (CD3^+^CD45RO^+^CD62L^-^) on a BD FACS Aria II and plated in X-VIVO medium (Gibco, USA) supplemented with 10% FBS (Gibco), at 37 °C and 5% CO2. TEM cells were activated with 10 ng/ml anti-CD3 monoclonal antibody (OKT3) (Invitrogen, USA) and 5 ng/ml anti-CD28 monoclonal antibody (CD28.2) (Invitrogen, USA) for 24 hours and washed. Then TEM cells were cultured with IL-7 (10 ng/ml) and IL-15 (20 ng/ml) (Peprotech, USA) with or without IL-2 (300 U/ml). Every 2–3 days medium and cytokines was replenished. TEM cells were collected after 5 days and phenotype was analyzed by multi-color flow cytometry, using the following antibodies: PE-Cy5-conjugated CD3 (UCHT1, Beckman Coulter, USA), PE-conjugated CD45RO (UCHL1, Beckman Coulter, USA), FITC-conjugated CD62L (DREG-56, Biolegend, USA), and PE-Cyanine7-conjugated PD-1 (A17188B, Biolegend, USA). Acquisition was performed on CytoFlex flow cytometry (Beckman Coulter). Data analyses were performed with CytExpert software. Pembrolizumab (anti-PD-1 Ab) (10 μg/ml, 24h, MedChemExpress, China) was added to build an *in vitro* anti-PD-1 therapy model when TEM cells were co-cultured with MKN45 cells at an effector-to-target (E: T) ratio of 5:1.

### Cytometric bead array analysis of cytokines

For a co-culture system, MKN45 cells were added on day 5 to different TEM culture groups, respectively (E: T ratio of 5:1) with or without Pembrolizumab. After 12 h, the concentration of IFN-γ in the culture supernatants were quantified using the Human IFN-γ Flex Set (Bead B8) (BD Bio-sciences). The samples were analyzed by CytoFlex flow cytometry (Beckman Coulter).

### Cytotoxicity assay

The cytotoxicity of different TEM culture groups was evaluated *in vitro* against MKN45 on day 5 at different E:T ratios at 37 °C and 5% CO2 for 12 hours using carboxy fluorescein succinimidyl amino ester and propidium iodide (CFSE/PI) assay. MKN45 cells were pre-labeled with 2.5 μM CFSE (Invitrogen, USA) at 37 °C in 5% CO_2_ for 20 min, followed by washing. Subsequently, different TEM culture groups and MKN45 cells were co-cultured at E:T ratios of 1:1, 2:1, 5:1, and 10:1 for 12 h. Cytotoxicity was assessed by PI (Beyotime, China) staining, with CFSE^+^ populations representing total target cells and CFSE^+^PI^+^ cells indicating dead targets. Cytotoxicity was calculated as the percentage of CFSE^+^PI^+^ over CFSE^+^ cells.

### Database analyses

We used the Human Protein Atlas (HPA) database (https://www.proteinatlas.org) by searching the term PDCD1 to profile the expression of PDCD1 in various immune cells. Immune cell infiltration analyses were performed between the samples from responders and non-responders to anti PD-1 immunotherapy on the basis of Immune Checkpoint Blockade therapy Atlas (ICBatlas) database (https://guolab.wchscu.cn/ICBatlas//#!/response) (19).

### Statistical analyses

All analyses were performed using IBM SPSS Statistic 26.0 (SPSS Inc, Chicago, IL, USA) and GraphPad Prism 8.0 (GraphPad Software, Inc, SigmaPlot). The chi-square test was used to compare categorical variables. Wilcoxon signed-rank test was used for nonparametric comparison among groups with different ICI responses. ROC (Receiver Operating Characteristic) curves were constructed to compare the performance of biomarkers in predicting ICI response. Survival analyses were estimated by the Kaplan-Meier analysis with the log-rank test. Univariate and multivariate statistical analyses of relevant variables associated with ICI therapy were evaluated by Cox proportional hazard models. Spearman correlation analysis was performed to test the correlation. Results were considered statistically significant at two- sided *P* values <0.05.

## Results

### Patient characteristics

**Figure 1:**
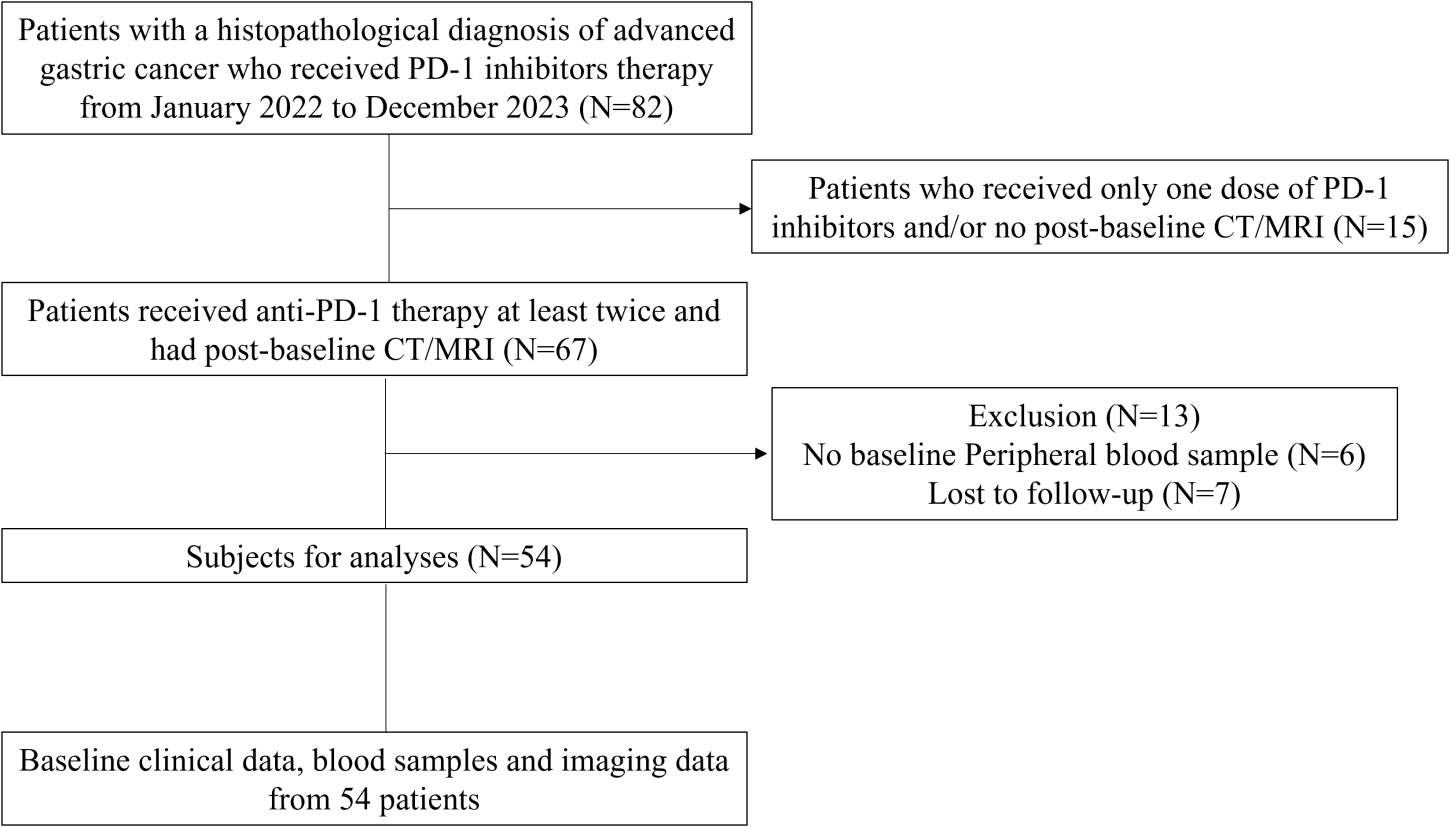
The flowchart of the enrollment process. CT, computed tomography; MRI, magnetic resonance imaging.

A total of 54 advanced GC patients treated with PD-1 inhibitors plus chemotherapy were included in the study and the baseline characteristics of the enrolled patients were summarized in **Table 1**. There were no differences in age, gender, metastasis, or other key variables between responders and non-responders, confirming that the groups were well-balanced and supporting the validity of our subsequent analyses. From January 2022 to December 2023, 54 patients with locally advanced or metastatic GC received at least two cycles of PD-1 inhibitors and underwent computed tomography (CT) imaging after the initiation of treatment at our institution (**Figure 1**). Follow up ended on December 31, 2023. Median patient age was 63 years (range 57–70 years) and 40 (74.1%) patients were men. Among the patients who could be evaluated for RECIST response, 25(46.3%) were PR, 18(33.3%) were SD for >6 months, and were defined as responders; 7(13.0%) were PD, 4(7.4%) were SD for <6 months, and were defined as non-responders. By December 2023, the median follow-up time was 12.9 months (range, 5.4–16.5 months). **Table 2** indicates immune-related adverse events (irAEs) in patients with GC on anti-PD-1 immunotherapy. The majority of irAEs observed were grade 1–2 in severity and were resolved with symptomatic treatment according to National Comprehensive Cancer Network guidelines for managing immunotherapy-related toxicities. Responders had significantly higher PD-L1 combined positive score (CPS) (22C3(DAKO)) (P = 0.016) compared to non-responders. There were no statistically significant differences in gender, age, metastasis, tumor grade, surgical treatment, tumor markers including carcinoembryonic antigen (CEA), carbohydrate antigen 199 (CA199) and carbohydrate antigen 724 (CA724) between responders and non-responders.

**Table 1:** Subgroup characteristics of patients with different efficacy from anti-PD-1 immunotherapy.

Characteristics	Responders (n=43)	Non-responders (n=11)	P
Median age, years(range)	65 (57-71)	60(54-64)	0.547
Gender, n (%)			0.909
Male	32 (74.4%)	8 (72.7%)	
Female	11 (25.6%)	3(27.3%)	
PD-L1CPS			
<5	14(32.6%)	8(72.7%)	0.016*
≥5	29(67.4%)	3(27.3%)	
Tumor grade, n (%)			0.350
Well	1 (2.3%)	0 (0.0%)	
Moderately	16 (37.2%)	6 (54.5%)	
Poorly	17 (39.6%)	5 (45.5%)	
Undetermined	9(20.9%)	0 (0.0%)	
Previous surgery, n (%)			0.564
Yes	5(11.6%)	2 (18.2%)	
No	38 (88.4%)	9 (81.8%)	
Metastatic status, n (%)			0.610
Yes	42 (97.7%)	11 (100.0%)	
No	1 (2.3%)	0 (0.0%)	
CEA, ng/ml, n (%)			0.696
≤ 10	30 (69.8%)	7 (63.6%)	
> 10	13 (30.2%)	4 (36.4%)	
CA199, u/ml, n (%)			0.841
≤ 27	21 (48.8%)	5 (45.5%)	
> 27	22 (51.2%)	6 (54.5%)	
CA724, u/ml, n (%)			0.196
≤ 6.9	18 (4.9%)	7 (663.6%)	
> 6.9	25 (58.1%)	4 (36.4%)	

**Table 2:** Summary of adverse events.

Adverse events	Grade 1 (%)	Grade 2 (%)	Grade 3 (%)	Grade 4 (%)
Anemia	9 (16.7)	6 (11.1)	2 (3.7)	1 (1.9)
Leukopenia	11 (20.4)	6 (11.1)	0 (0.0)	0 (0.0)
Neutropenia	3 (5.6)	6 (11.1)	0 (0.0)	0 (0.0)
Thrombocytopenia	5 (9.3)	3 (5.6)	3 (5.6)	0 (0.0)
Pyrexia	1 (1.9)	1 (1.9)	0 (0.0)	0 (0.0)
Cough	0 (0.0)	0 (0.0)	0 (0.0)	0 (0.0)
Nausea or emesis	1 (1.9)	0 (0.0)	0 (0.0)	0 (0.0)
Diarrhea	1 (1.9)	0 (0.0)	0 (0.0)	0 (0.0)
Rash	3 (5.6)	4 (7.4)	1 (1.9)	0 (0.0)
Nephritis	0 (0.0)	0 (0.0)	0 (0.0)	0 (0.0)
Myocarditis	0 (0.0)	0 (0.0)	0 (0.0)	0 (0.0)
Hyperthyroidism	0 (0.0)	0 (0.0)	0 (0.0)	0 (0.0)
Hepatitis	0 (0.0)	1 (1.9)	0 (0.0)	0 (0.0)
Sum	34(63.0)	27(50.0)	6(11.1)	1 (1.9)

### Effector memory T cells exhibited the highest PD-1 expression across memory T cell subsets

**Figure 2:**
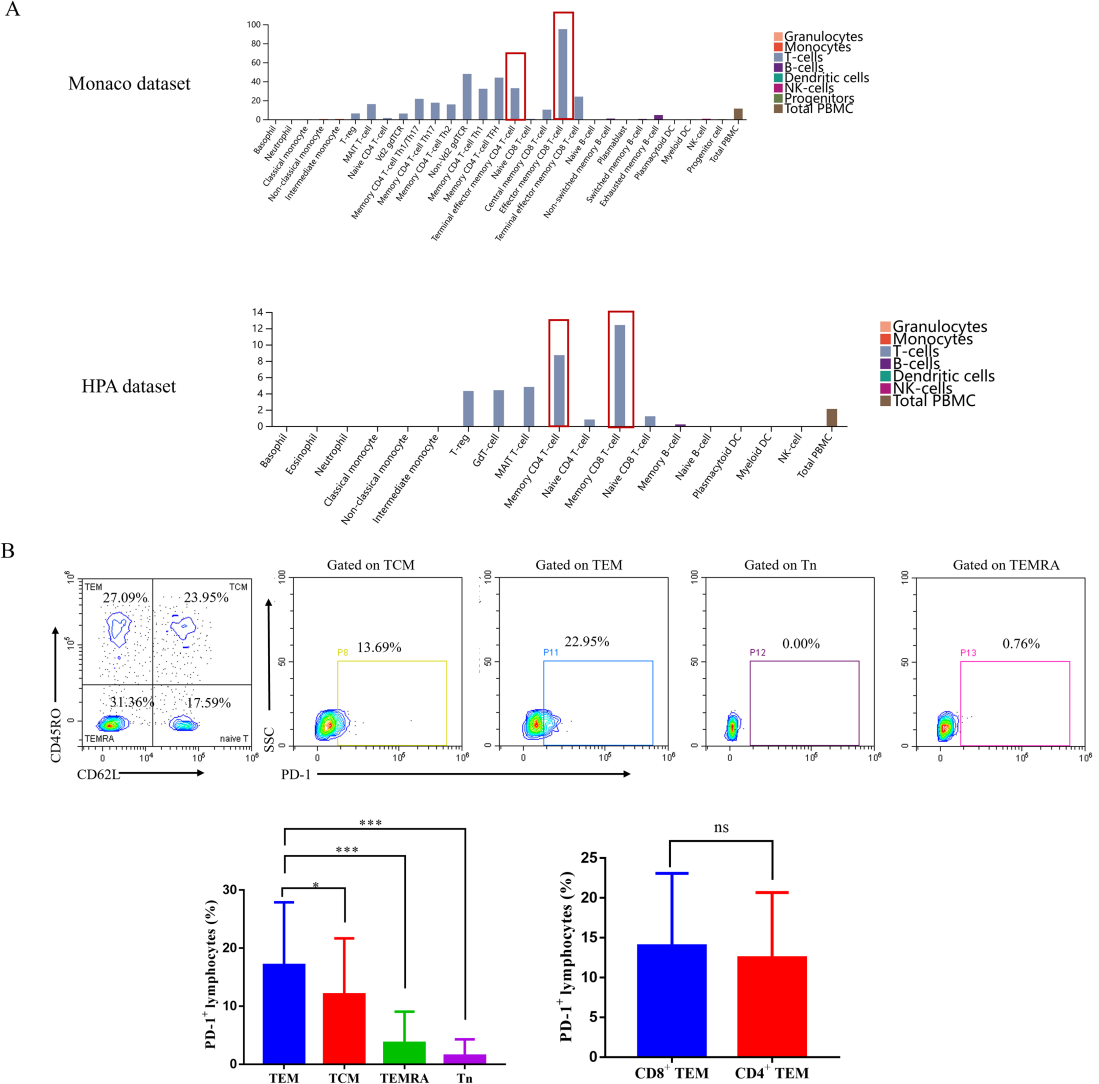
Effector memory T cells exhibited the highest PD-1 expression across memory T cell subsets in peripheral blood. **(A)** PD-1 expression of different immune cells in the blood in the HPA dataset and Monaco dataset. **(B)** The average percentage of PD-1^+^ cells in peripheral memory T lymphocytes in 54 advanced gastric cancer patients. Representative flow cytometry countor plots (the top of B) and summation data plots (the bottom of B). HPA, Human Protein Atlas. ***P < 0.001, *P < 0.05, ns, no significant.

To elucidate comprehensive immunological features in circulation, we subjected 54 pretreatment blood samples from patients with typical clinical courses (responders: PR or SD≥6 months; non-responders: SD<6 months or PD) to flow cytometry. Multiple immune parameters such as PD-1 expression in tumor infiltrating lymphocytes (TILs) and peripheral blood mononuclear cells (PBMC) have been described in human and were proposed as predictive of anti-PD-1 clinical response (20–22). We utilized HPA database to retrieve and integrated the results from two databases: the HPA dataset and Monaco scaled dataset. The analysis found that PD-1 is mainly expressed on memory T cells (**Figure 2A**). We profiled the PD-1 expression landscape in human memory T cells (**Figure 2B**). Central memory T cells (TCM) are characterized as CD45RO^+^CD62L^+^, effector memory T cells (TEM) are identified as CD45RO^+^CD62L^-^, naive T cells (Tn) are characterized as CD45RO^-^CD62L^+^, and terminally differentiated effector memory T cells (TEMRA) are identified as CD45RO^-^CD62L^-^. Analyses of 54 peripheral blood samples revealed that PD-1 was predominantly expressed on TEM, albeit at varied levels across samples (TEM vs TCM, *P* < 0.05; TEM vs TEMRA, *P* < 0.001; TEM vs Tn, *P* < 0.001), and PD-1 expression was higher in CD8^+^ TEM compared to CD4^+^ TEM, but the difference was not statistically significant between the two subsets (*P*>0.05).

### Responders have high percentage of PD-1^+^ cells in effector memory T cells in peripheral blood

**Figure 3:**
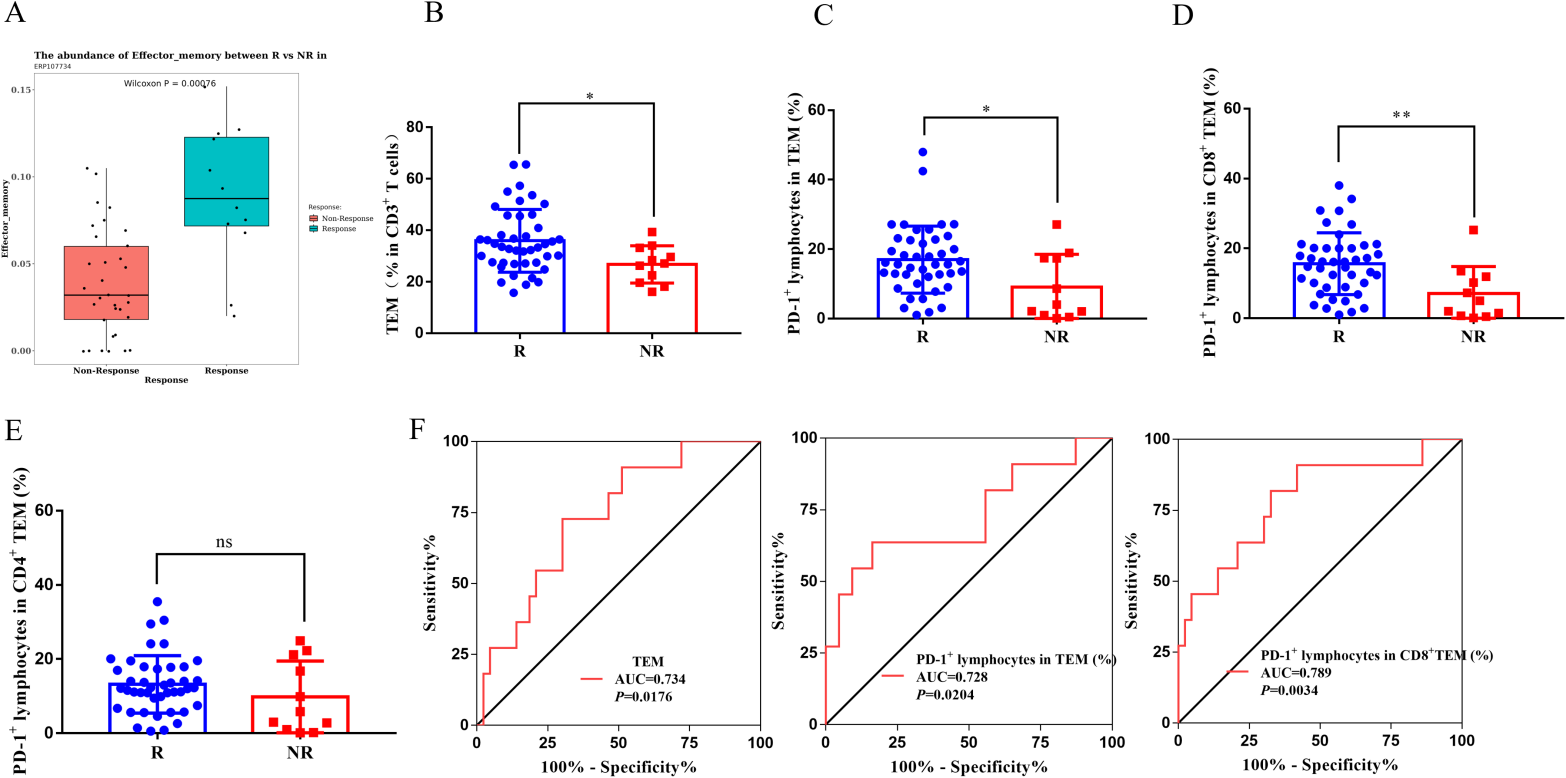
Responders have high percentage of PD-1^+^ cells in effector memory T cells in peripheral blood. **(A)** Abundances of TEM between R and NR to anti-PD-1 therapy in the ICBatlas dataset. The average percentage of **(B)** TEM in peripheral CD3^+^ T lymphocytes, **(C)** PD-1^+^ lymphocytes in TEM, **(D)** CD8^+^ TEM and **(E)** CD4^+^ TEM between R and NR in our enrolled 54 advanced gastric cancer patients. **(F)** ROC curves analyses for the optimal cut-off values of TEM, percentage of PD-1^+^ lymphocytes in TEM and CD8^+^ TEM, respectively. TEM, effector memory T cells; ICBatlas, Immune Checkpoint Blockade therapy Atlas; R, Responders; NR, Non-responders. **P < 0.01, *P < 0.05, ns, no significant.

Data within ICBatlas associated with immunotherapy response revealed that higher abundances of TEM in responders to anti-PD-1 therapy versus non-responders (**Figure 3A**) in GC. In our 54 enrolled patients, TEM (not CD4^+^ TEM or CD8^+^ TEM, **Supplementary Figure S1**) was significantly higher in the peripheral blood from responders than in those from non-responders (**Figure 3B**, P < 0.05). It was previously reported that PD-1 expression on CD8^+^ T cells with memory-like properties in the tumor were associated with benefit of patients treated with monoclonal antibody to PD-1 (16, 23). Therefore, we aimed to investigate whether percentage of PD-1^+^ cells in TEM in the circulation could serve as a predictive marker for PD-1 blockade therapies. Our analysis of the 54 GC patients included revealed that the percentage of PD-1^+^ cells in TEM is significantly higher in responders compared to non-responders (**Figure 3C**, P < 0.05). Moreover, our results show that responders have higher percentage of PD-1^+^ cells in CD8^+^ TEM (**Figure 3D**, P < 0.01) rather than in CD4^+^ TEM (**Figure 3E**, *P*>0.05).

### Determination of optimal cut−off values for effector memory T cells and percentage of PD-1^+^ cells in effector memory T cells

As revealed in **Figure 3F**, the areas under ROC curves (AUC) for TEM in peripheral CD3^+^ T cells, percentage of PD-1^+^ lymphocytes in TEM and CD8^+^ TEM were 0.734, 0.728 and 0.789, respectively. The optimal cut-off values for TEM in peripheral CD3^+^ T cells, percentage of PD-1^+^ lymphocytes in TEM and CD8^+^ TEM were 29.75%, 8.66% and 12.17%, respectively. Patients were separately divided into high and low groups based on the optimal cut-off values.

### Patients with high percentage of PD-1^+^ cells in effector memory T cells have better survival outcomes and prognostic value in comparison with other blood-based biomarkers

**Figure 4:**
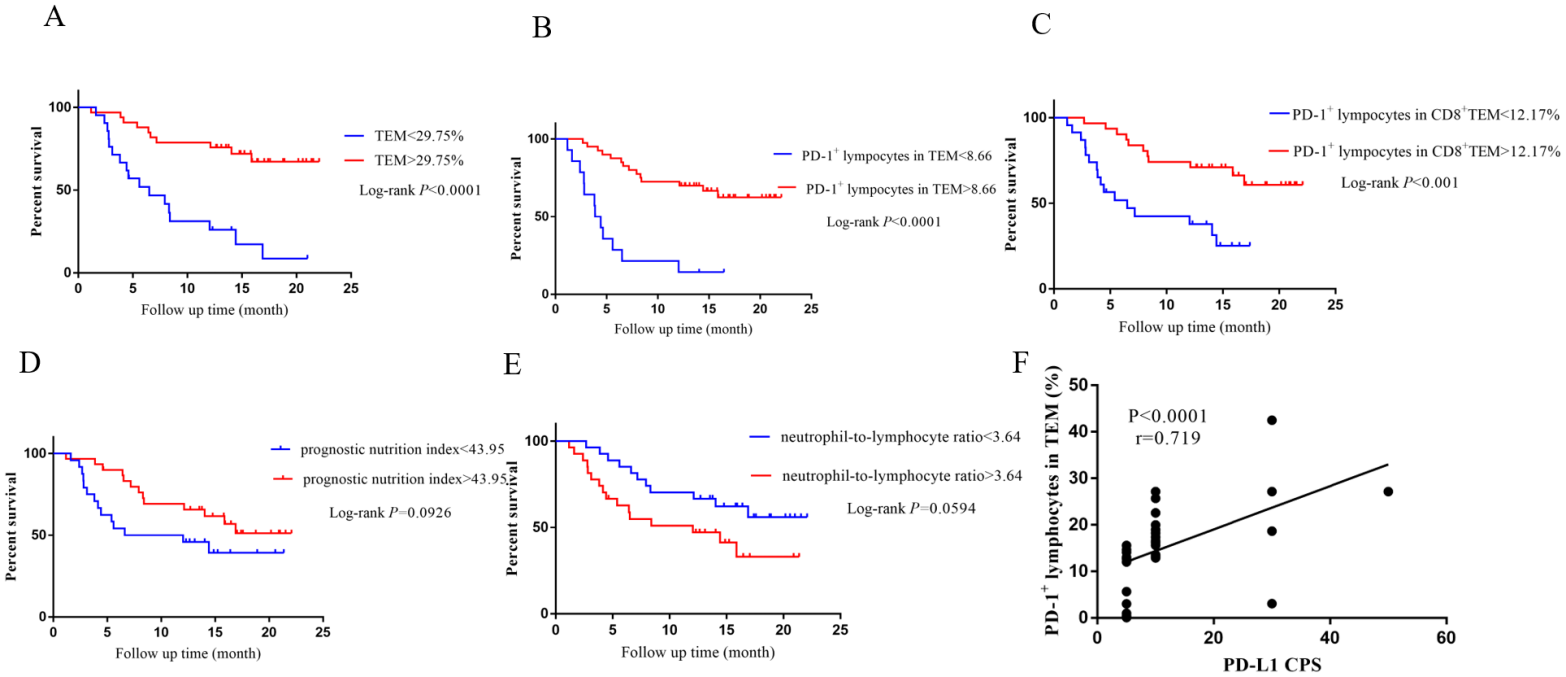
Patients with high percentage of PD-1^+^ cells in effector memory T cells have better survival outcomes and prognostic value in comparison with other blood-based biomarkers. Kaplan-Meier curves for PFS in patients with values above or below **(A)** TEM, PD-1^+^ lymphocytes in **(B)** TEM and **(C)** CD8^+^ TEM, **(D)** PNI as well as **(E)** NLR cutoff in 54 advanced gastric cancer patients. **(F)** Spearman correlation analysis between PD-1^+^ lymphocytes in TEM and PD-L1 CPS. Abbreviations: TEM, effector memory T cells; PNI, prognostic nutrition index; NLR, neutrophil-to-lymphocyte ratio.

Blood-based biomarkers, such as the neutrophil-to-lymphocyte ratio (NLR), prognostic nutrition index (PNI), have been reported to be associated with the efficacy of anti-PD1 therapy (24). Patients were stratified as low-and high-NLR (<3.64 and ≥3.64, respectively), low-and high-PNI (<43.95 and ≥43.95) groups. The grouping criteria were selected according to their optimal cut-off values in our 54 GC patients (**Supplementary Figure S2**). Over the course of follow-up for the 54 patients enrolled in our study, only three patients experienced a fatal outcome. Consequently, we opted to use PFS instead of OS as the primary endpoint for our statistical analysis. Patients with high percentage of TEM (1-year PFS: high vs. low = 75.76 vs. 25.97%, *P* < 0.0001; **Figure 4A**), PD-1^+^ lymphocytes in TEM (1-year PFS: high vs. low = 70.00 vs. 14.29%, *P* < 0.0001 **Figure 4B**) and CD8^+^ TEM (1-year PFS: high vs. low = 70.97 vs. 37.68%, *P* < 0.001 **Figure 4C**) at the baseline indicated a good prognosis with significantly prolonged PFS. PNI ≥ 43.95 (1-year PFS: high vs. low = 65.77 vs. 45.83%, *P* = 0.0926 **Figure 4D**) and NLR ≤ 3.64 (1-year PFS: high vs. low = 47.06 vs. 66.67%, *P* = 0.0594 **Figure 4E**) were not associated with significantly prolonged PFS. The prognostic values of TEM and percentage of PD-1^+^ lymphocytes in TEM and CD8^+^ TEM were further assessed by Cox proportional hazards models. The results of the univariate and multivariate analyses of PFS were showed in **Table 3**. Univariate analyses presented that high PD-L1 CPS (*P* < 0.05), high percentage of TEM (*P* < 0.01), high percentage of PD-1^+^ lymphocytes in TEM (*P* < 0.001) and CD8^+^ TEM subsets (*P* < 0.01), as well as low NLR (*P* < 0.05) prior to treatment were associated with prolonged PFS. Factors which were statistically significant in the univariate analysis and PNI were incorporated into the multivariate analyses. In the multivariate analyses, six independent Cox models were separately constructed. Each model included only one parameter. The multivariate analyses indicated that PD-L1 CPS (*P* < 0.05) and percentage of PD-1^+^ lymphocytes in TEM (*P* < 0.01) were independent prognostic factors for survival times in advanced GC patients receiving chemoimmunotherapy. Miao−Zhen Qiu et al. demonstrated that PD-L1 CPS ≥5 is significantly associated with improved response and extended PFS in advanced gastric or gastroesophageal junction adenocarcinoma patients treated with a combination of PD-1 inhibitors and chemotherapy (25). Therefore, we sought to analyze whether there is a correlation between percentage of PD-1^+^ lymphocytes in TEM and the CPS score in patients with PD-L1 CPS ≥ 5. As shown, we identify a positive correlation between PD-L1 CPS and PD-1^+^ lymphocytes in TEM using spearman correlation analysis (**Figure 4F** P < 0.0001, r=0.719). In summary, percentage of PD-1^+^ lymphocytes in TEM in circulation is superior to other blood-based indicators as predictive biomarkers for PFS after administration of PD-1 inhibitors.

**Table 3:** Univariate and multivariate analyses of progression-free survival in advanced gastric cancer patients receiving immunotherapy and chemotherapy.

Variable	Univariate analysis		Multivariate analysis	
	HR (95% CI)	P−value	HR (95% CI)	P−value
Age (≥ 60vs.< 60 years)	1.112 (0.510-2.424)	0.789		
Sex (male vs. female)	0.573 (0.216-1.524)	0.265		
Differentiation (well or moderate vs. poor)	0.567 (0.248-1.295)	0.178		
PD-L1CPS (< 5 vs. ≥ 5)	0.359 (0.164-0.786)	0.010	0.274 (0.101-0.739)	0.011
CEA (≤ 10 vs. > 10)	1.630 (0.748-3.553)	0.219		
CA199 (≤ 27 vs. > 27)	1.298 (0.596-2.829)	0.511		
CA724 (≤ 6.9 vs. > 6.9)	0.786 (0.364-1.695)	0.539		
TEM (< 29.75 vs. ≥ 29.75)	0.300 (0.132-0.685)	0.004	0.565 (0.219-1.462)	0.239
PD-1^+/^TEM (< 8.66 vs. ≥ 8.66)	0.176 (0.079-0.390)	<0.001	0.191 (0.065-0.560)	0.003
PD-1^+^/CD8^+^ TEM (< 12.17 vs. ≥ 12.17)	0.318(0.145-0.699)	0.004	0.809(0.280-2.340)	0.696
NLR	2.584(1.148-5.816)	0.022	1.744(0.605-5.627)	0.303
PNI	0.500(0.230-1.084)	0.079	1.247(0.440-3.533)	0.678

### PD-1 blockade restores anti-tumor activity of TEM cells *in vitro*

**Figure 5:**
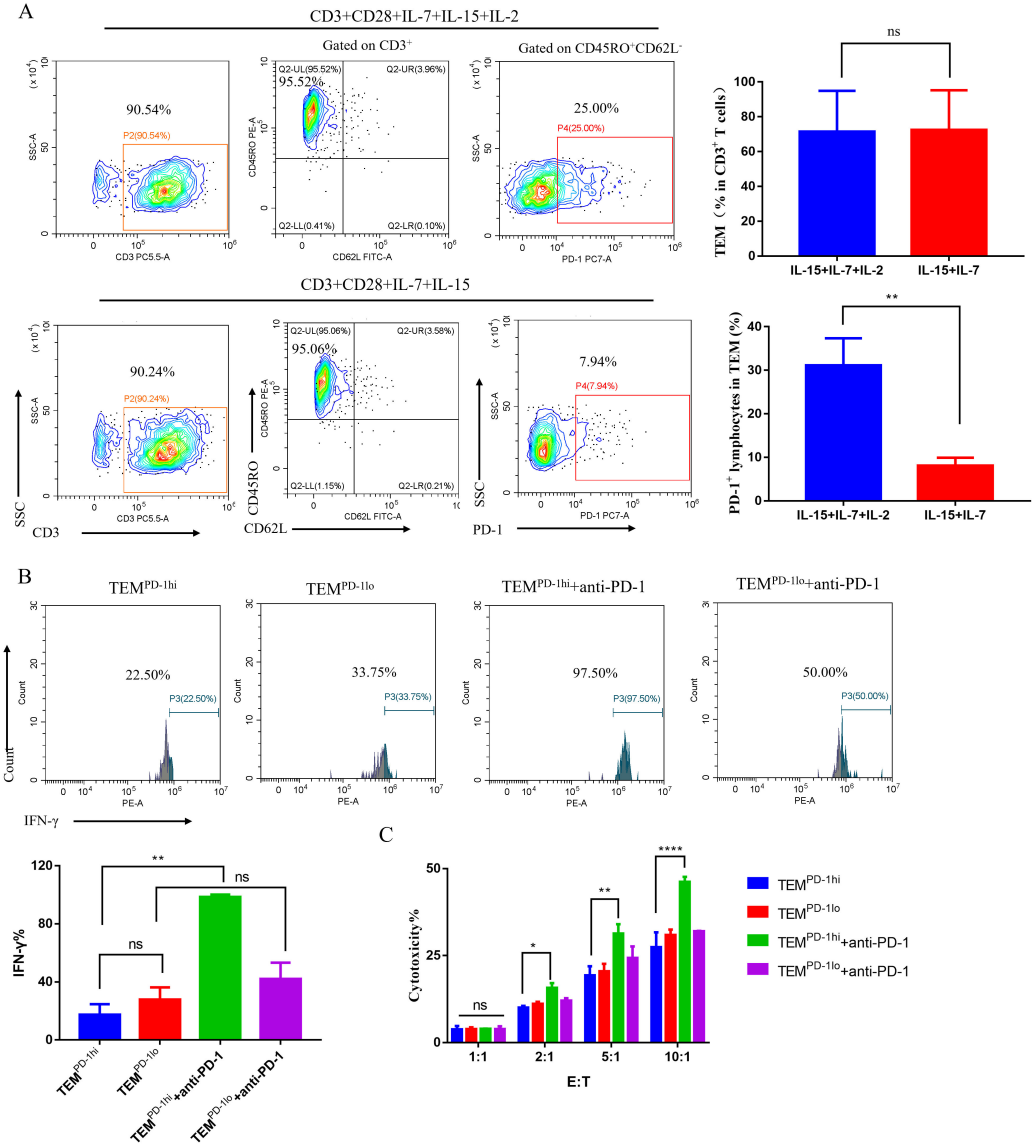
PD-1 blockade restores anti-tumor activity of PD-1^+^TEM cells *in vitro*. **(A)** TEM, PD-1^+^ lymphocytes in TEM in different culture methods at day 5. Representative flow cytometry countor plots (the left of A) and summation data plots (the right of A). **(B)** Comparison of IFN-γ secretion of TEM PD-1hi and TEM PD-1lo following incubation with MKN45 at designated E: T ratio of 5:1, Representative flow cytometry histogram (the top of B) and summation data plots (the bottom of B). **(C)** The cytotoxic reactivity of TEM ^PD-1hi^ and TEM ^PD-1lo^ was measured using CFSE/PI cytotoxicity assay, the target cells were MKN45. Abbreviations: TEM, effector memory T cells; TEM^PD-1hi^, TEM cells with high PD-1 expression; TEM ^PD-1lo^, TEM cells with high low expression. The error bars represented mean±SEM (n=3, biologically independent samples); statistical significance was determined by two-tailed unpaired Student’s t-tests.****P <0.0001, **P <0.01, *P <0.05, ns, no significant.

In our above-mentioned research, we found that high percentage of PD-1^+^ lymphocytes in TEM is associated with better efficacy and prognosis of chemo-immunotherapy combinations. In order to further confirm the relationship between high percentage of PD-1^+^ lymphocytes in TEM and the efficacy of anti-PD-1 therapy, we designed an *in vitro* anti-PD-1 treatment model. Requirements for the generation of memory T cells are CD3/CD28 engagement and culture with IL-7 and IL-15 (26) and high dose IL-2 leads to upregulation of PD-1. We used flow cytometry to sort TEM cells from PBMCs and first stimulated sorted TEM cells with low-dose anti-CD3/CD28 monoclonal antibody and then one group was stimulated with IL-15 and IL-7 and IL-2, and another group was stimulated with IL-15 and IL-7. After 5 days, we found that the proportion of TEM cells was similar between the groups (**Figure 5A** top, *P*>0.05), but the TEM cells from the IL-2 stimulation group had higher PD-1 expression (**Figure 5A** bottom, P < 0.01). Therefore, we named TEM cells cultured with IL-15 and IL-7 and IL-2 as TEM ^PD-1hi^; TEM cells cultured with IL-15 and IL-7 as TEM ^PD-1lo^ for the subsequent PD-1 blockade experiment. TEM cells with high PD-1 expression (TEM ^PD-1hi^) and low PD-1 expression (TEM ^PD-1lo^) were co-cultured with MKN45 cells at an E:T ratio of 5:1 for 12 hours, with or without the addition of anti-PD-1 Ab. The concentration of IFN-γ in the culture supernatants were quantified. The results showed that the addition of anti-PD-1 Ab significantly restored the IFN-γ secretion capacity of TEM ^PD-1hi^ (**Figure 5B****f5**, P < 0.01), and this was significantly higher than TEM ^PD-1lo^. In contrast, the IFN-γ secretion capacity of TEM ^PD-1lo^ did not show a significant increase upon the addition of the anti-PD-1 Ab. A cytotoxicity assay involving CFSE-labeled MKN45 cells and TEM ^PD-1hi^/TEM ^PD-1lo^ with or without the addition of anti-PD-1 Ab was conducted for a duration of 12 h. Our results showed that the addition of anti-PD-1 Ab significantly increased the cytotoxicity of the TEM ^PD-1hi^ group against MKN45 at E:T ratios of 2:1, 5:1, and 10:1, with the most significant effect observed at 10:1. However, the addition of anti-PD-1 Ab did not significantly enhance the killing effect of the TEM ^PD-1lo^ (**Figure 5C**, 2:1 *P* < 0.05; 5:1 *P* < 0.01; 10:1 *P* < 0.0001). These results suggested that anti-PD-1 Ab can restore the anti-tumor activity of TEM cells with high expression of PD-1.

## Discussion

A substantial proportion of patients lack sufficient tissue at diagnosis to perform standard clinical testing, prompting the suggestion to search for predictive biomarkers in peripheral blood to categorize patients who may benefit from ICI therapy plus chemotherapy. Several candidate blood biomarkers include serum lactate dehydrogenase (LDH) levels, serum thyroid peroxidase antibody (A-TPO), serum T3 (FT3), serum cytokines, blood cell ratios, serum tumor markers and immune-cell subsets (27–29). A focus of blood-based analyses of immune cell subsets lies on peripheral PD-1^+^ T cells (30). In particular, functional subtypes within the CD8^+^PD-1^+^ T-cell compartment are thought to predict responses to PD-1 blockade in lung cancer, while CD4^+^ PD-1^+^ T cells have been reported as non-responders to PD-1 blockade in malignant melanoma (31, 32). However, the potential roles of PD-1 expression across memory T-cell subsets remain inadequately explored. Unlike general CD8^+^ or CD4^+^ T cells, PD-1 expression may reflect exhaustion or activation; PD-1 on TEM cells specifically marks a subset with enhanced functional potential for tumor eradication. For example, Lai et al. reported that PD-1^+^ CD8^+^ T cells within PBMCs from ovarian cancer patients correlated with improved survival, but these populations were heterogeneous, comprising naive and central memory subsets (33). Additionally, CD4^+^PD-1^+^ T cells may include suppressive regulatory T cells, potentially linked to resistance to PD-1 blockade. Nishikawa et al. emphasized that a higher ratio of PD-1^+^ Tregs to PD-1^+^ CD8^+^ T cells in the tumor microenvironment signals adverse responses to PD-1 blockade, because PD-1^+^ Tregs exhibit enhanced immunosuppression upon PD-1 inhibition (18). By focusing on TEM cells (which exclude most Tregs), we can isolate a functionally committed population that directly contributes to tumor control and minimize confounding effect of Tregs.

It has been demonstrated that TRM infiltration in the tumor immune microenvironment and circulating T cells mount recall responses during ICB therapy (34), suggesting the significance of investigating memory T cells. Memory is the hallmark of the adaptive immune system, protective memory is facilitated by TEM, which migrate to inflamed peripheral tissues and exhibit immediate effector functions. In contrast, reactive memory is mediated by TCM, which home to T cell zones of secondary lymphoid organs. While TCM have little direct effector function, they readily proliferate and differentiate into effector cells upon encountering cognate antigen (35). Many studies have investigated PD-1 expression on memory T cells, as well as their characteristic features and functions. For example, high PD-1 expression on TRM reflects tissue residence, rather than exhaustion and these TRM exhibited decreased cytolytic capacity, potentially benefiting from ICB therapy (36). Besides, a study by Chauvin et al. showed that T cell immunoreceptors with Ig and ITIM domains (TIGIT)^+^ PD-1^+^ tumor antigen-specific CD8^+^ T cells from patients with melanoma displayed an effector memory phenotype and responded well to PD-1 targeted therapies (37). Therefore, we hypothesize that TEM, which can rapidly produce effector cytokines to mediate protective immunity, may have their cytokine secretion function impaired due to PD-1 expression. This suggests that they may be particularly responsive to PD-1 targeted therapies.

Here, we show that percentages of PD-1 expression in TEM in the peripheral blood in responder groups were significantly higher than that in non-responder groups. The high percentages of circulating PD-1^+^ TEM at the baseline were significantly associated with good prognosis in advanced GC patients treated with ICI therapy plus chemotherapy. Since the patients we included received chemo-immunotherapy combinations, we further constructed an *in vitro* anti-PD-1 monotherapy model. We cultured patient PBMCs-derived TEM cells as the *in vitro* model for subsequent experiments. Interestingly, we were pleased to find that the anti-tumor activity of the TEM ^PD-1hi^ group was significantly increased after the addition of anti-PD-1 Ab. The *in vitro* results further validated the potential of using the percentages of PD-1 expression on TEM cells as a predictive biomarker for the efficacy of anti-PD-1 therapy. Our PD-1^+^ TEM cell marker could complement existing prediction models by adding a functional dimension that is easily accessible from blood. For instance, while TMB reflects neoantigen load, PD-1^+^ TEM cells may indicate the capacity of the immune system to respond to these antigens. This is especially relevant in chemo-immunotherapy combinations, where chemotherapy may enhance antigen release and TEM recruitment.

PD-L1 CPS has shown better enrichment for efficacy of checkpoint inhibitors than tumor cell PD-L1 expression in advanced GC (38). However, abundant variety of PD-L1 antibodies such as Dako 22C3, Dako 28–8 and Ventana SP-142 used in IHC assay has posed difficulties in establishing a unified scoring standard for clinical evaluation guide (39). In our study, we found a positive correlation between PD-L1 CPS and percentages of circulating PD-1^+^ TEM using Spearman correlation analysis. Moreover, peripheral blood samples are more readily accessible compared to tissue samples, and flow cytometric detection is easier to repeat dynamically in clinical practice and is more amenable to standardization than immunohistochemistry. These findings suggest the potential utility of percentages of PD-1^+^ lymphocytes in TEM as a predictive biomarker for the efficacy of PD-1 immunotherapy.

Nevertheless, some limitations in our study. Firstly, our study was based on a relatively small sample size (n=54) from a single institution, and the missing information and data from patients lost to follow-up may have limited the statistical power and the generalizability of our findings. Furthermore, the limited number of outcome events precluded a robust analysis of OS, leading us to rely on PFS as the primary endpoint. Therefore, future studies involving larger, multi-center prospective cohorts are essential to validate the predictive power of PD-1^+^ TEM cells and to establish clinically applicable cutoff values. Additionally, extended follow-up in a larger population will be necessary to confirm the long-term prognostic value of this biomarker and its association with OS. Considering the accessibility and ease of standardization of peripheral blood samples render the assessment percentages of PD-1^+^ lymphocytes in TEM highly promising as a potential biomarker in predicting efficacy of PD-1 immunotherapy.

## Conclusions

In conclusion, our findings suggest that the percentages of PD-1^+^ lymphocytes in TEM in peripheral blood can effectively predict the response and prognosis to PD-1 immunotherapy in patients with advanced GC. Due to the availability of peripheral blood samples, it can also be used clinically to select patients who are likely to benefit from PD-1 immunotherapy in future.

## Data Availability

The original contributions presented in the study are included in the article/**Supplementary Material**. Further inquiries can be directed to the corresponding authors.

## Supplementary material

Supplementary Figure 1 The average percentage of TEM in peripheral **(A)** CD4^+^ and **(B)** CD8^+^ T lymphocytes in advanced gastric cancer patients. TEM, effector memory T cells.

Supplementary Figure 2 ROC curves analyses for the optimal cut-off values of neutrophil-to-lymphocyte ratio, and prognostic nutrition index in advanced gastric cancer patients.
