# Supplementary figures and images for: Circulating PD-1+ effector memory T cells predict anti-PD-1 efficacy in advanced gastric cancer
Source: Front Immunol. 2025 Dec 10;16:1720724. doi: 10.3389/fimmu.2025.1720724 (PMC12727954; doi:10.3389/fimmu.2025.1720724)

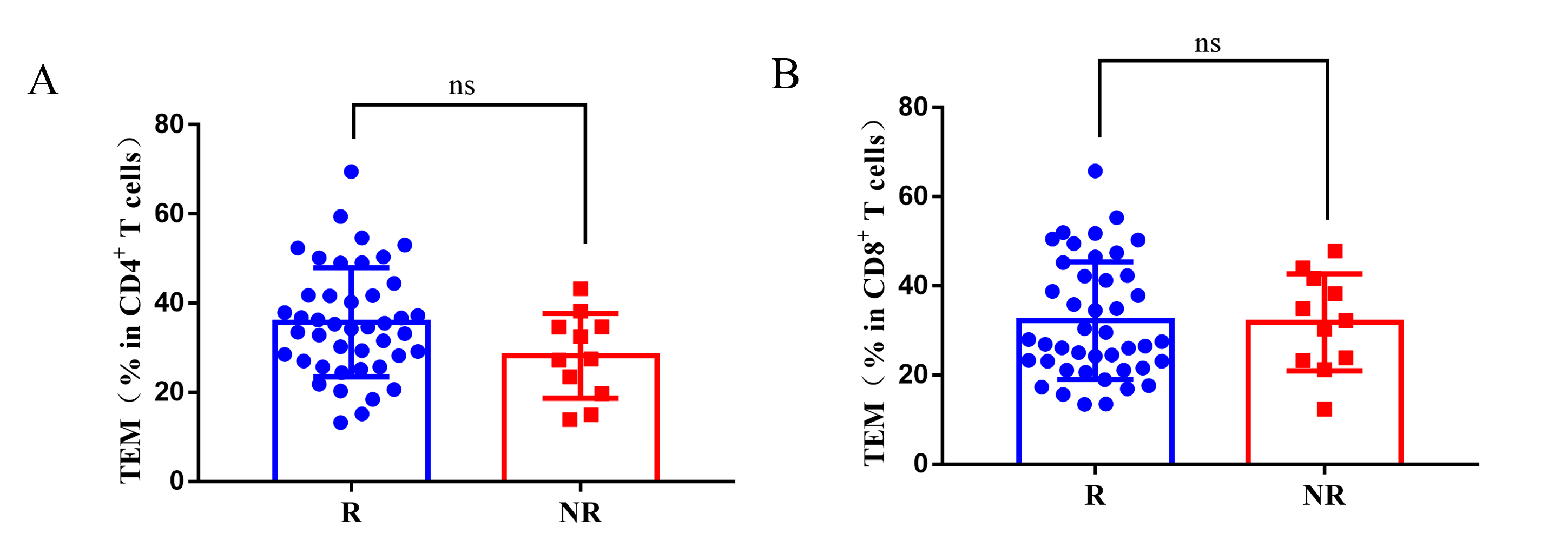

Supplement: Supplementary Figure 1 — The average percentage of TEM in peripheral (A) CD4+ and (B) CD8+ T lymphocytes in advanced gastric cancer patients. TEM, effector memory T cells. [file Image1.tif]

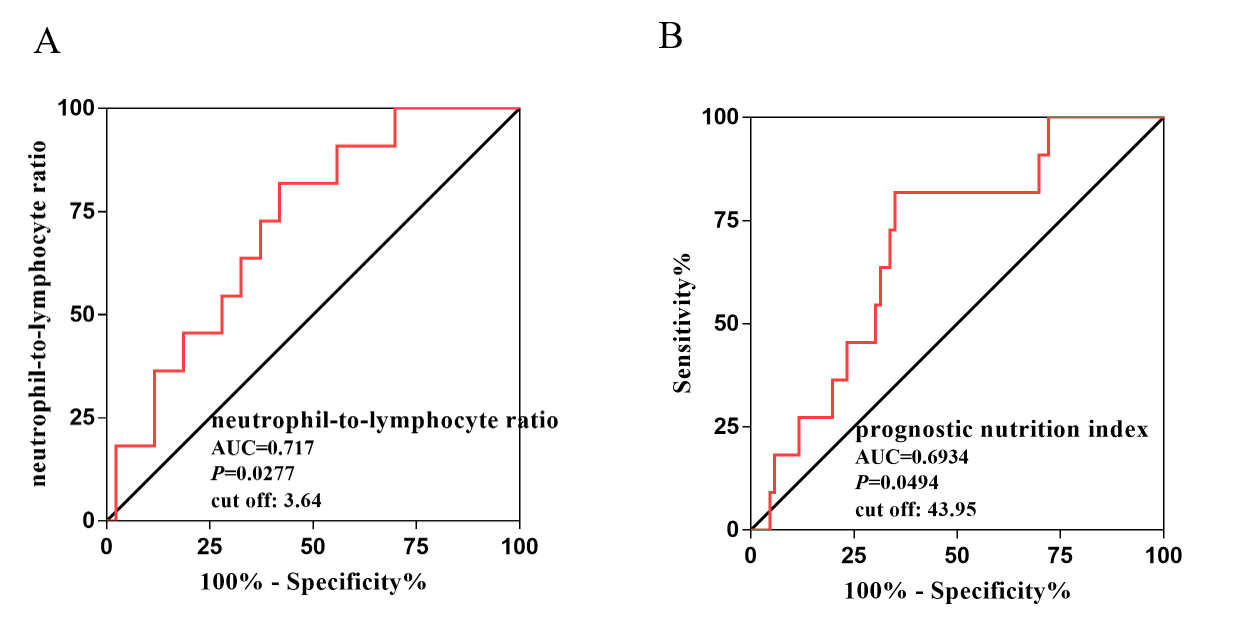

Supplement: Supplementary Figure 2 — ROC curves analyses for the optimal cut-off values of neutrophil-to-lymphocyte ratio, and prognostic nutrition index in advanced gastric cancer patients. [file Image2.tif]
